# Inhibition of cannabinoid degradation enhances hippocampal contextual fear memory and exhibits anxiolytic effects

**DOI:** 10.1016/j.isci.2024.108919

**Published:** 2024-01-15

**Authors:** Jinming Zhang, Junmin Zhang, Ruiqi Yuan, Wenxin Han, Yuan Chang, Lingyang Kong, Chunling Wei, Qiaohua Zheng, Xingchao Zhu, Zhiqiang Liu, Wei Ren, Jing Han

**Affiliations:** 1 Key Laboratory of Modern Teaching Technology, Ministry of Education, Shaanxi Normal University, Xi’an 710000, China; 2 Faculty of Education, Shaanxi Normal University, Xi’an 710000, China; 3 Department of Histology and Embryology, School of Basic Medical Science, Xi’an Medical University, Xi’an 710000, China; 4 Heze Hospital of Traditional Chinese Medicine, Heze 274000, China

**Keywords:** Biological sciences, Neuroscience, Behavioral neuroscience, Cognitive neuroscience

## Abstract

Recent studies have demonstrated the pivotal involvement of endocannabinoids in regulating learning and memory, but the conclusions obtained from different paradigms or contexts are somewhat controversial, and the underlying mechanisms remain largely elusive. Here, we show that JZL195, a dual inhibitor of fatty acid amide hydrolase and monoacylglycerol lipase, can enhance the performance of mice in a contextual fear conditioning task and increase the time spent in open arms in the elevated zero maze (EZM). Although the effect of JZL195 on fear memory could not be inhibited by antagonists of cannabinoid receptors, the effect on the EZM seems to be mediated by CB1R. Simultaneously, hippocampal neurons are hyperactive, and theta oscillation power is significantly increased during the critical period of memory consolidation upon treatment with JZL195. These results suggest the feasibility of targeting the endocannabinoid system for the treatment of various mental disorders.

## Highlights

• Systemical administration of JZL195 promotes contextual fear memory
• Inhibition of cannabinoid degradation exhibits anxiolytic effects
• Inhibition of cannabinoid degradation enhances hippocampal activity
• The anxiolytic effect of JZL195 depends on CB1R

## Graphical abstract

**Figure:**
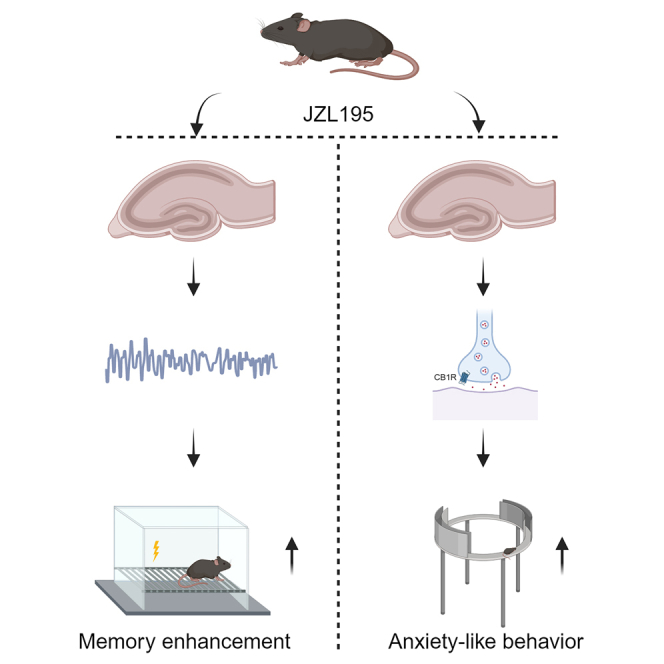

## Introduction

Memory deficits are prominent features of many mental disorders, such as anxiety,^1^ insomnia,^2^ and even Alzheimer’s disease (AD).^3^ In recent decades, the development of various drugs that were anticipated to alleviate memory deficits but that eventually showed limited clinical benefits has been observed.^3^ As a widely distributed neuromodulatory system, the endocannabinoid system (ECS) and its relative chemical compounds are involved in virtually all brain functions through either traditional cannabinoid receptor type 1 (CB1R) and type 2 (CB2R) or other G protein-coupled receptors (GPRs).^4^ Retrograde control of synaptic transmission and plasticity is a powerful way through which the ECS regulates brain functions;^5^ hence, the ECS may serve as a therapeutic target in neurological disorders. CB1R is the key mediator of endocannabinoid-elicited short- and long-term synaptic plasticity, which is regarded as the neurological basis of learning and memory, in the hippocampus, prefrontal cortex, and amygdala.^6^^,^^7^ Although sparsely expressed in the central nervous system (CNS), CB2Rs are also indispensable for memory recall.^8^ Traditional cannabinoids (e.g., Δ^9^-tetrahydrocannabinol, THC) have been demonstrated to trigger memory impairment^9^ and an increased incidence of mental diseases because of their psychotropic properties.^10^ In this regard, cannabidiol (CBD), a nonpsychotropic *Cannabis*-derived compound, has been found to improve the performance of mice in memory-retrieval tasks^11^^,^^12^ and has been applied in registered clinical trials for several diseases.^10^^,^^13^

The concentrations of endocannabinoids are collaboratively controlled by enzymes that catalyze the synthesis and hydrolysis of these metabolites. In particular, the hydrolysis of endocannabinoids, 2-arachidonoyl glycerol (2-AG) and anandamide (AEA), is catalyzed preferably by monoacylglycerol lipase (MAGL) and fatty acid aminohydrolase (FAAH), respectively.^14^ Generally, inhibition of MAGL and FAAH causes an accumulation of 2-AG and AEA within synapses, leading to the reinforcement of cannabinoid-induced intracellular signaling and synaptic plasticity. Global MAGL knockout promotes hippocampus-dependent spatial memory recall in mice in a water maze test.^15^ However, dual ablation of MAGL and FAAH through pharmacological inhibition or genetic manipulation results in a THC-like detrimental effect on memory, as evaluated by the performance of mice in the water maze task.^16^ Recently, several reviews have addressed the role of the ECS as a new target for treating neurodegenerative disorders by alleviating either inflammatory responses or synaptic plasticity in the hippocampus.^17^^,^^18^^,^^19^

To pharmacologically inhibit MAGL and FAAH individually or in combination, JZL184 (target to MAGL), URB597 (or PF-3845, target to FAAH), and JZL195 (target to both MAGL and FAAH) are often used. JZL184 typically inhibits MAGL and then results in an increased level of 2-AG, but at a high dose (40 mg/kg) it may also inhibit FAAH.^16^ In contrast to JZL184 and URB597, JZL195 seems to work in a more complex way. JZL195 has also been demonstrated to modulate neuroinflammation,^20^ locomotion,^21^ pain,^22^ and self-grooming^23^ through CB1Rs across species under certain circumstances. Although the role of the single drugs JZL184 and URB597 in fear conditioning and extinction has been investigated,^24^ we failed to search the literature in PubMed about JZL195 when using keywords such as “JZL195,” “fear,” and “fear memory,” alone or combined. Therefore, showing the effect and underlying mechanism of JZL195 on fear memory, in terms of its potential as a clinical drug when targeting various mental disorders, is essential.

Due to these inconsistent results, it is of great necessity to probe the role of JZL195 in contextual fear memory formation and anxiety-like behavior. To address these issues, we used fear conditioning and the elevated zero maze (EZM) to assess fear memory and anxiety-like behavior, immunofluorescence and electrophysiology to assess neuronal activity, and pharmacology and *trans*-genetic mouse lines to assess the role of CB1R and CB2R. Overall, we found that the administration of JZL195, a dual inhibitor of FAAH and MAGL, could promote contextual fear memory consolidation and recall and exhibited anxiolytic effects.

## Results

### Inhibition of cannabinoid hydrolysis was anxiolytic and enhanced contextual but not cued fear memory

**Figure 1:**
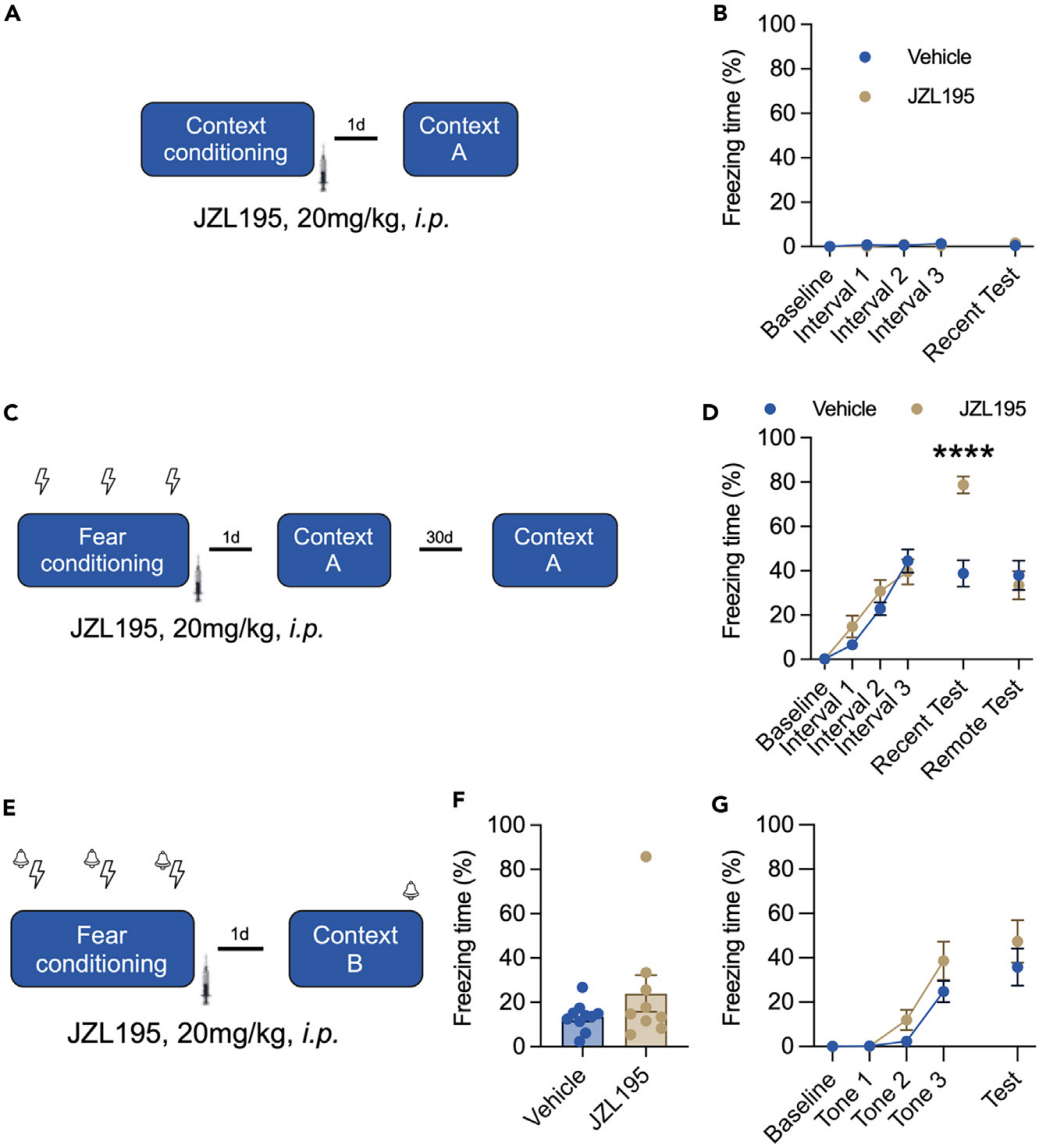
JZL195 promotes contextual but not cued fear memory consolidation (A) The procedure of naive contextual conditioning (n_Vehicle_ = 10, n_JZL195_ = 8). (B) The freezing time of mice in Context A (two-way ANOVA, the main effect of intervals: *F*_(4,64)_ = 2.182, p = 0.081; the main effect of vehicle vs. JZL195: *F*_(1,16)_ = 0.0003, p = 0.986; the interaction effect: *F*_(4,64)_ = 2.114, p = 0.089; Sidak’s post hoc test of vehicle vs. JZL195 at recent test: p = 0.248). (C) The procedure of contextual fear conditioning. (D) Freezing time of mice during fear conditioning, recent contextual test, and remote contextual test (two-way ANOVA, the main effect of intervals: *F*_(5,155)_ = 62.850, p < 0.0001; the main effect of Vehicle (n = 18) vs. JZL195 (n = 15): *F*_(1,31)_ = 3.076, p = 0.089; the interaction effect: *F*_(5,155)_ = 9.789, p < 0.0001; Sidak’s post hoc test of Vehicle vs. JZL195 at Recent Test: p < 0.0001, and at Remote Test: p = 0.985). (E) The procedure of cued fear conditioning. (F) The basal contextual freezing in Context B (Mann-Whitney test, *U*_(91,99)_ = 36, p = 0.497). (G) Freezing time of mice during tone-cued fear conditioning and tone-cued test (two-way ANOVA, main effect of tones: *F*_(4,68)_ = 29.340, p < 0.0001; main effect of vehicle (n = 10) vs. JZL195 (n = 9): *F*_(1,17)_ = 3.383, p = 0.083; interaction effect: *F*_(4,68)_ = 0.868, p = 0.488; Sidak’s post hoc test of vehicle vs. JZL195 at test: p = 0.475). Data are represented as the mean ± SEM. “∗∗∗∗” represents p < 0.0001.

**Figure 2:**
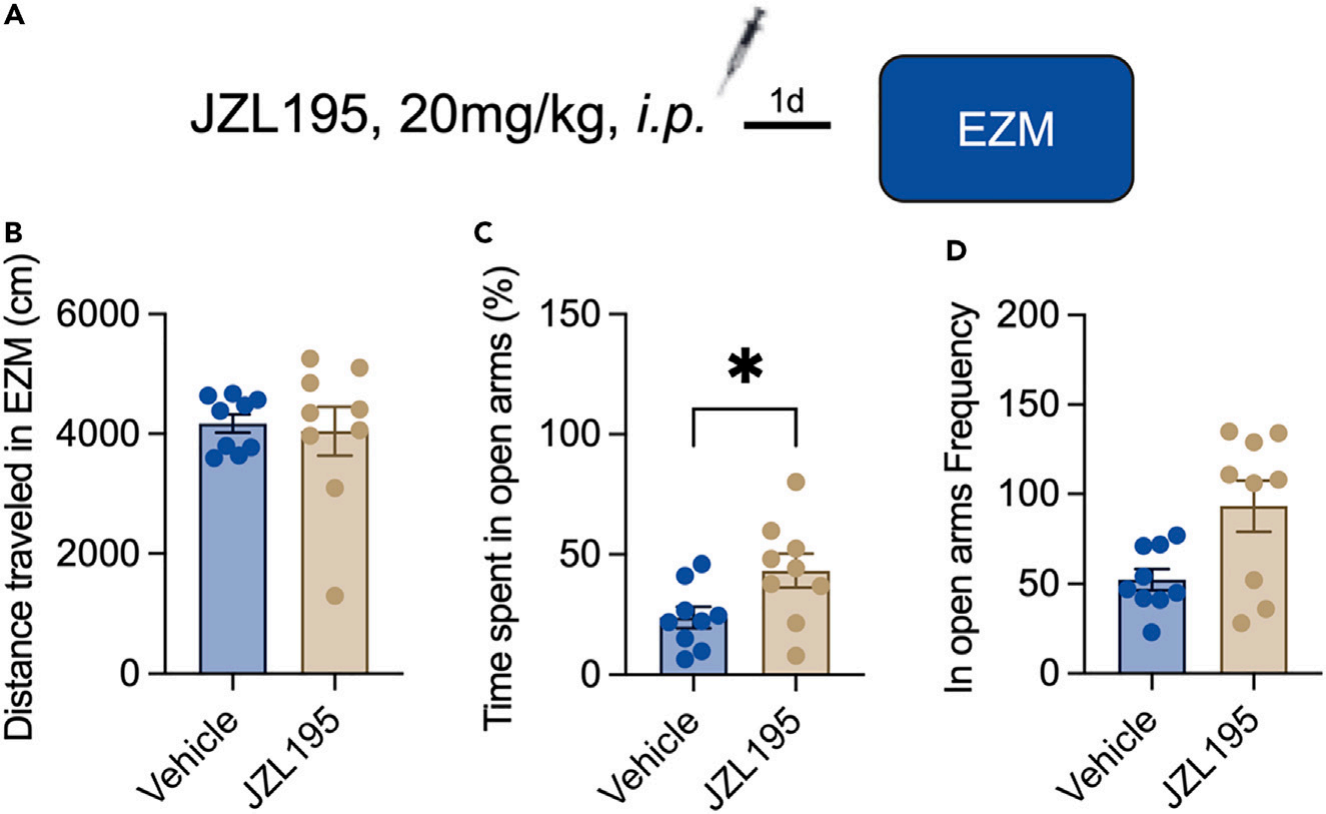
JZL195 is anxiolytic one day after injection (A) The procedure of the elevated zero maze test (n = 9/group). (B) The distance that mice moved in the EZM (Mann-Whitney test, *U*_(82,89)_ = 37, p = 0.796). (C) The time that mice spent in the open arms of the EZM (Student’s *t* test, *t*_(16)_ = 2.346, p = 0.032). (D) The frequency of mice entering the open arms of the EZM (Mann-Whitney test, *U*_(65,106)_ = 36, p = 0.077).

Fear memory has been studied in both cue-dependent and context-dependent fear conditioning paradigms. We found that systemic administration of JZL195 did not change the basal freezing time of mice in the absence of foot shocks in contextual conditioning recall (Figures 1A and 1B) and also the intact short-term memory (Figure S1). Then, we employed a contextual fear conditioning paradigm for the test. Compared with that of untreated mice, those treated with JZL195 displayed significantly prolonged freezing time on Day 2 in the recent recall test, and this increasing effect was abolished in the remote recall test (Figure 1D). Next, we explored the effect of JZL195 on cue fear conditioning paradigms. However, we failed to detect a significant difference between vehicle- and JZL195-treated mice in the cued fear memory recall test (Figures 1E–1G) or basal contextual freezing in Context B (Figure 1F). Mice also spent much more time in the open arms of the EZM (Figure 2), with no modification of traveling distance (Figure 2B). These results showed that JZL195 promotes contextual fear memory consolidation and recall but not cued fear memory.

### JZL195 enhanced c-Fos expression induced by the contextual fear memory test

**Figure 3:**
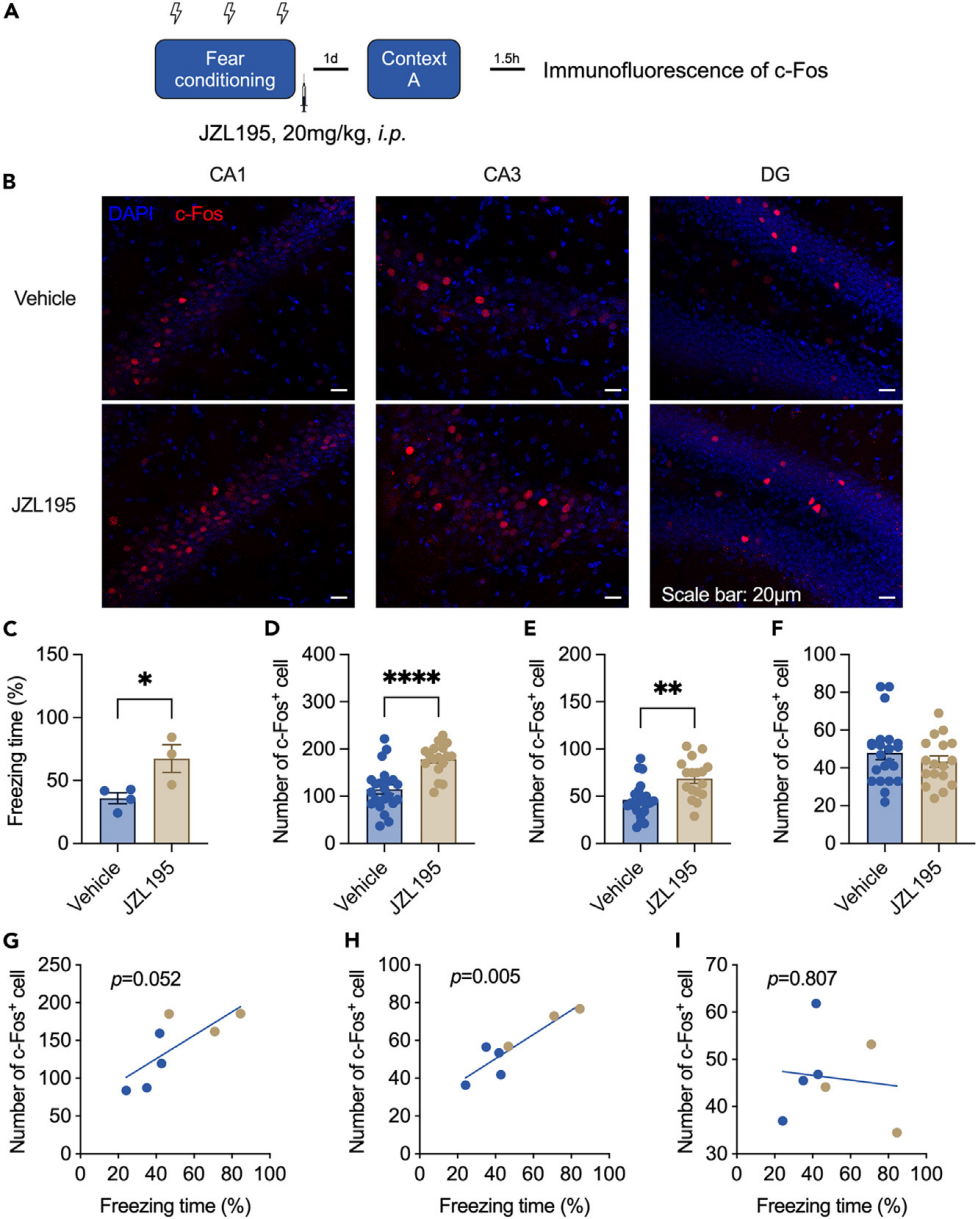
c-Fos expression in the hippocampus was increased after the contextual test (A) Immunofluorescence experiment procedure. (B) Representative images of c-Fos expression in CA1 (Left), CA3 (Middle), and DG (Right). (C) The freezing time of mice that were used to detect c-Fos expression in the recent test (unpaired t test, *t*_(5)_ = 2.980, p = 0.031). (D) The number of c-Fos-positive cells in the CA1 area of the hippocampus (unpaired t test, *t*_(37)_ = 4.800, p < 0.0001). (E) The number of c-Fos-positive cells in the CA3 area of the hippocampus (unpaired t test, *t*_(37)_ = 3.564, p = 0.001). (F) The number of c-Fos-positive cells in the DG area of the hippocampus (unpaired t test, *t*_(37)_ = 0.953, p = 0.347). (G) Correlation analysis between the number of c-Fos-positive cells in the CA1 area of the hippocampus and freezing time (F test, *F*_(1,5)_ = 6.440, p = 0.052). (H) Correlation analysis between the number of c-Fos-positive cells in the CA3 area of the hippocampus and freezing time (F test, *F*_(1,5)_ = 23.860, p = 0.005). (I) Correlation analysis between the number of c-Fos-positive cells in the DG area of the hippocampus and freezing time (F test, *F*_(1,5)_ = 0.067, p = 0.807). n = 22 slices from 4 vehicle-treated mice and n = 18 slices from 3 JZL195-treated mice. Data are represented as the mean ± SEM. ∗p < 0.05, ∗∗p < 0.01, ∗∗∗∗p < 0.0001.

We then tested whether increased freezing could activate the hippocampus, which plays a critical role in contextual fear conditioning. The procedure of the experiment is shown in Figure 3A, and a representative image of c-Fos expression is shown in Figure 3B (Left: Cornu ammonis 1 (CA1), Middle: Cornu ammonis 3 (CA3), Right: Dentate gyrus (DG)). The number of c-Fos-positive cells was higher in the hippocampal CA1 region of JZL195-treated mice than in vehicle-treated mice (Figure 3D). The hyperactivity of neurons was also found in the CA3 (Figure 3E) but not the DG (Figure 3F) region. Correlation analysis also showed that freezing time had a positive correlation with the number of c-Fos^+^ cells in CA1 (Figure 3G) and CA3 (Figure 3H) but not in the DG (Figure 3I). These data showed that the improvement in contextual fear memory consolidation and recall induced by JZL195 was accompanied by an increase in neuronal activation in the hippocampus.

### JZL195 enhanced hippocampal theta oscillation power in CA1 and neuronal activity

**Figure 4:**
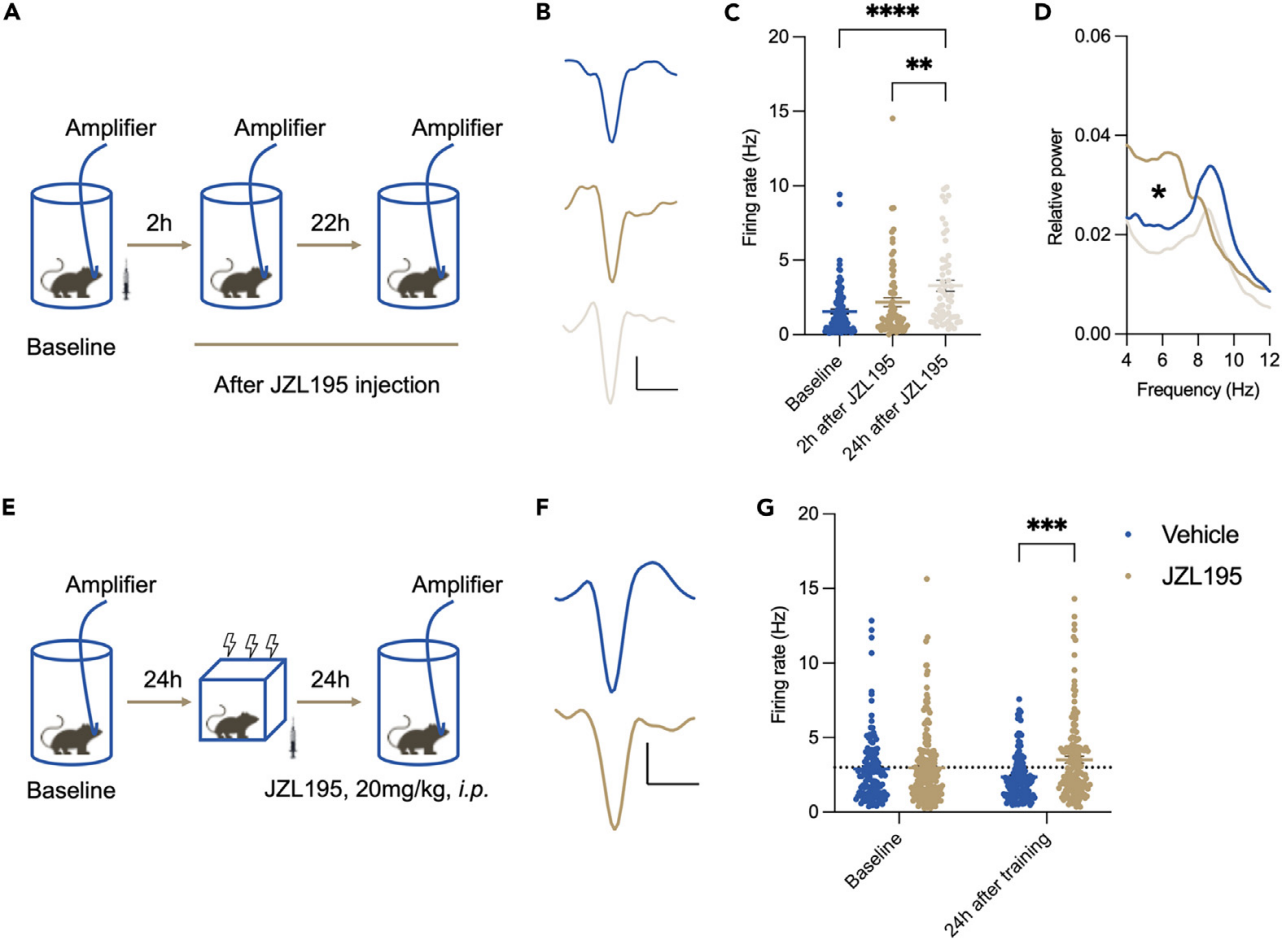
JZL195 enhanced theta oscillation power and hippocampal neuronal activity (A) The procedure of *in vivo* recording (results shown in B‒D). (B) Illustration of isolated single units in the experiment described in (A). Scale bar: 500 μs, 50 μA. (C) Firing rate of all isolated single units (Kruskal‒Wallis test, *KW*_(3)_ = 22.13, p < 0.0001; Dunn’s post hoc test: Baseline vs. 24 h after JZL195, p < 0.0001, 2 h after JZL195 vs. 24 h after JZL195, p = 0.002). (D) Power spectrum analysis of theta oscillation (Kruskal‒Wallis test, *KW*_(3)_ = 59.24, p < 0.0001; Dunn’s post hoc test: Baseline vs. 2 h after JZL195, p < 0.0001, Baseline vs. 24 h after JZL195, p = 0.043, 2 h after JZL195 vs. 24 h after JZL195, p < 0.0001). (E) The procedure of *in vivo* recording (results shown in F and G). (F) Illustration of isolated single units in the experiment described in (E). Scale bar: 500 μs, 50 μA. (G) Firing rate of all isolated single units (two-way ANOVA, main effect of test point: *F*_(1,591)_ = 0.0002, p = 0.989; main effect of vehicle vs. JZL195: *F*_(1,591)_ = 10.580, p = 0.001; interaction effect: *F*_(1.591)_ = 7.559, p = 0.006; Tukey’s post hoc test: baseline, vehicle vs. JZL195: p = 0.999; 24 h after JZL195, vehicle vs. JZL195: p = 0.0002). For subpanels A–D, n = 103, 73, and 55 from 2 to 3 mice, Baseline vs. 2 h after JZL195 vs. 24 h after JZL195; For subpanels E–G, in baseline, n = 128 and 182 from 4 mice, Vehicle vs. JZL195; in 24 h after training, n = 139 and 146 from 4 mice, Vehicle vs. JZL195. Data are represented as the mean ± SEM. ∗p < 0.05, ∗∗p < 0.01, ∗∗∗p < 0.001, ∗∗∗∗p < 0.0001.

Two hours after drug injection, the local field potential (LFP) data revealed a significant enhancement in the relative power of theta oscillations in CA1 of JZL195-treated mice (Figure 4D). Meanwhile, the single-unit activity measurement did not show any difference compared to the baseline (Figure 4C). Twenty-four hours later, a statistically significant increase in single-unit activity was measured in JZL195-treated mice (Figures 4A–4C). Contextual fear conditioning was then introduced. Although mice from both groups showed similar CA1 neuronal activity, the firing rate of all isolated single units recorded in JZL195-treated mice was significantly increased (Figures 4E‒4G). These data collectively suggested that enhanced activities in the hippocampus were induced by JZL195.

### Excitatory synaptic transmission was changed in CA1 pyramidal neurons

**Figure 5:**
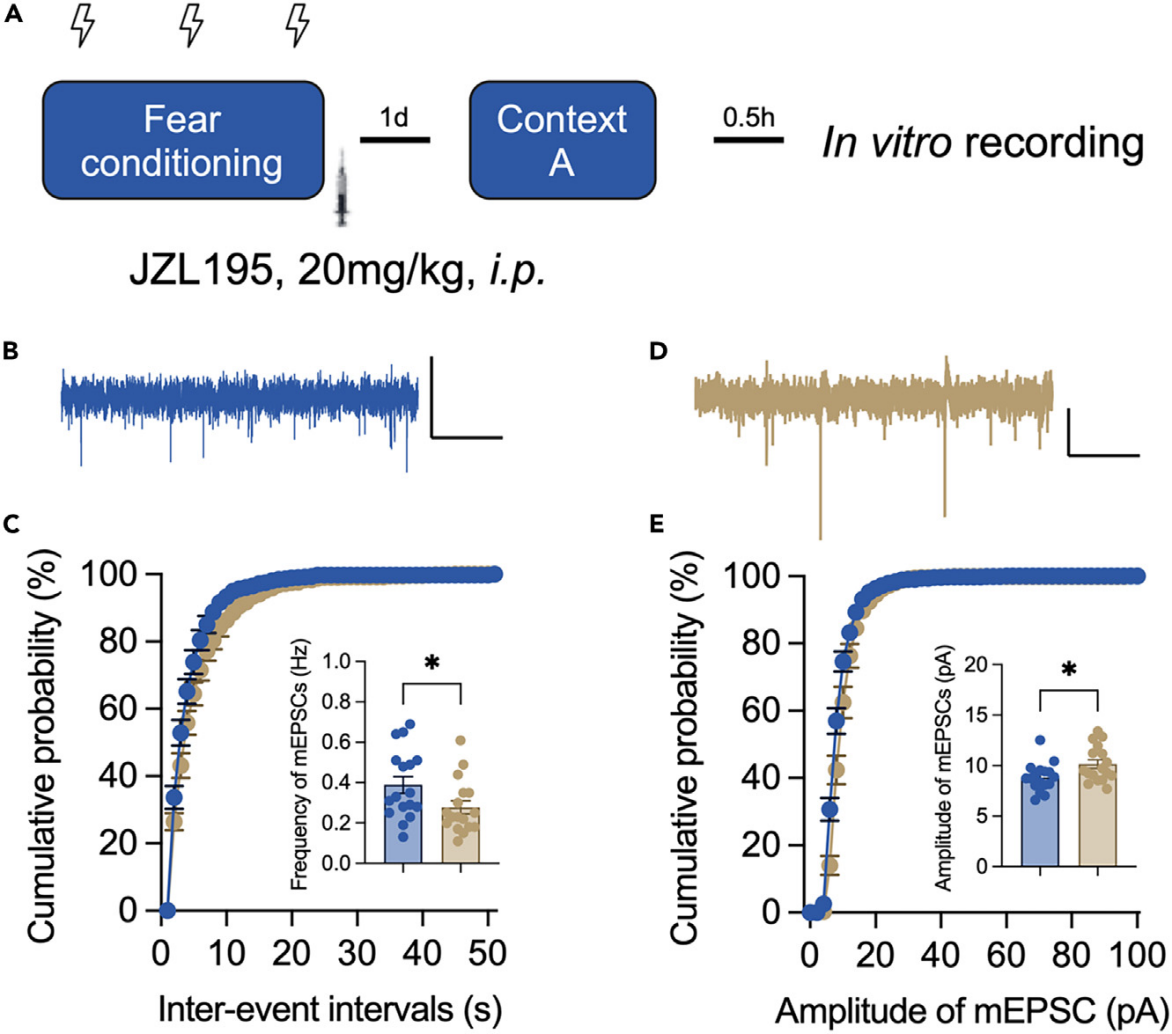
Frequency and amplitude of mEPSCs in CA1 pyramidal neurons after contextual test with or without JZL195 treatment (A) The procedure of *in vitro* recording (n = 17/group). (B) Illustration of mEPSCs recorded from vehicle-treated mice. Scale bar: 2 s, 10pA. (C) Cumulative probability of interevent intervals and the average frequency of mEPSCs (for cumulative probability analysis, Kolmogorov-Smirnov test, *D* = 0.333, p = 0.007; for average frequency comparison, unpaired t test, *t*_(32)_ = 2.130, p = 0.041). (D) Illustration of mEPSCs recorded from JZL195-treated mice. Scale bar: 2 s, 10pA. (E) Cumulative probability and the mean value of mEPSC amplitude (for cumulative probability analysis, Kolmogorov-Smirnov test, *D* = 0.392, p < 0.001; for average amplitude comparison, unpaired t test, *t*_(32)_ = 2.425, p = 0.021). Data are represented as the mean ± SEM. ∗p < 0.05.

Miniature excitatory postsynaptic current (mEPSC) was thus recorded in the CA1 pyramidal neurons (Figure 5A). Compared to those from vehicle-treated mice, neurons from JZL195-treated mice exhibited a decreased frequency of mEPSCs (Figures 5B and 5C) and an elevated average amplitude (Figures 5D and 5E). These results indicated that the intrahippocampal circuit may contribute to the increased neuronal activity in the CA1 area after JZL195 treatment.

### Hippocampus-specific knockout of CB1R blocked the anxiolytic effect of JZL195 but not fear memory recall

As shown in Figures S2A and S2B, intraperitoneal injection at doses of 0.03 and 3 mg/kg did not influence the performance of mice in the contextual fear conditioning task. Neither AM281 and NESS0327 nor SR144528 could counteract the effect of JZL195 (Figures S3A‒S3D). Although increased freezing time was observed in all of the JZL195-treated mice, it did not tend to be decreased by the coinjection of the antagonist of CB1R or CB2R.

**Figure 6:**
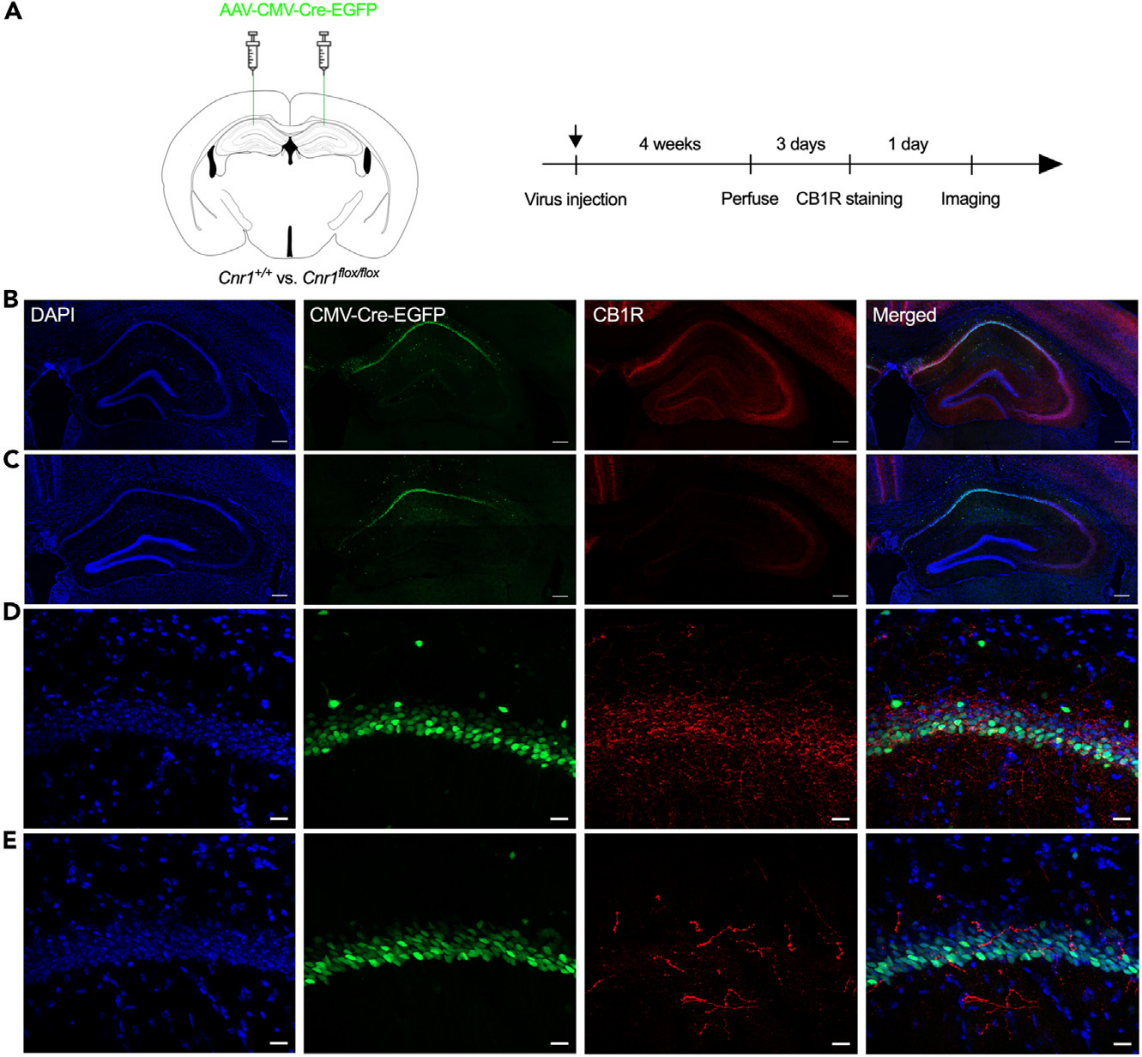
Construction and verification of the hippocampus-specific deletion of CB1R (A) The scheme of the virus injection (Left) and the timeline of the experiment (Right). (B) Representative image of the CB1R-stained hippocampus from wild-type mice (scale bar: 200 μm). (C) Representative image of the CB1R-stained hippocampus from CB1R^H−KO^ mice (scale bar: 200 μm). (D) Enlarged representative image of the CB1R-stained hippocampus from wild-type mice (scale bar: 20 μm). (E) Enlarged representative image of the CB1R-stained hippocampus from CB1R^H−KO^ mice (scale bar: 20 μm).

**Figure 7:**
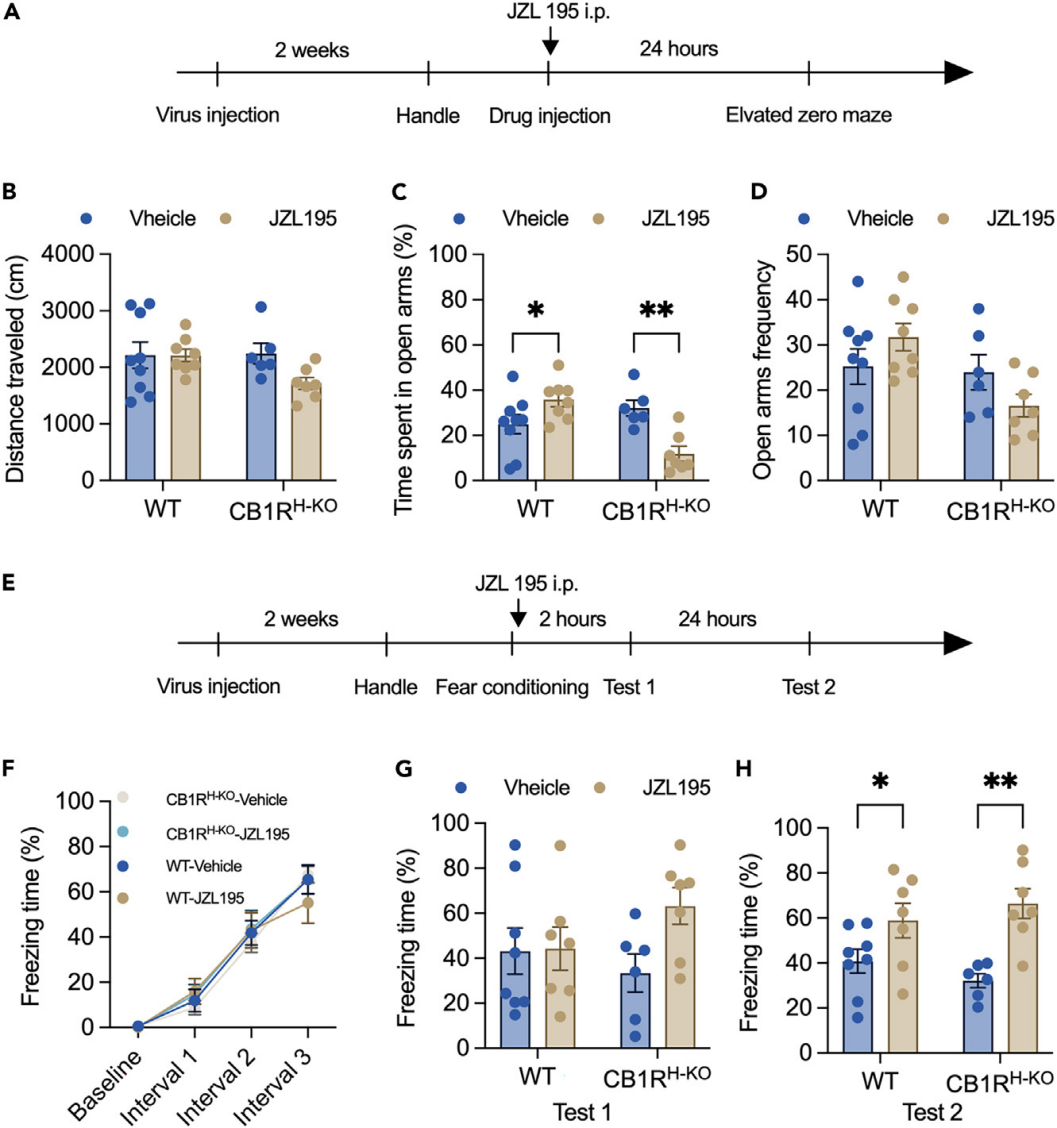
Hippocampus-specific knockout of CB1R blocked the anxiolytic effect of JZL195 but not fear memory recall (A) The procedure of virus injection and the EZM test (n = 6–9/group). (B) The distance that mice moved in the EZM (two-way ANOVA, main effect of gene type: *F*_(1,26)_ = 1.755, p = 0.197; main effect of vehicle vs. JZL195: *F*_(1,26)_ = 2.249, p = 0.146; interaction effect: *F*_(1,26)_ = 2.188, p = 0.151). (C) The time that mice spent in the open arms of the EZM (two-way ANOVA, main effect of gene type: *F*_(1,26)_ = 5.091, p = 0.033; main effect of vehicle vs. JZL195: *F*_(1,26)_ = 1.617, p = 0.215; interaction effect: *F*_(1,26)_ = 17.520, p = 0.0003; Sidak’s post hoc test of WT-Vehicle vs. WT-JZL195: p = 0.036, CB1R^H−KO^-Vehicle vs. CB1R^H−KO^-JZL195: p = 0.003). (D) The frequency of mice entering the open arms of the EZM (two-way ANOVA, main effect of gene type: *F*_(1,26)_ = 5.495, p = 0.027; main effect of vehicle vs. JZL195: *F*_(1,26)_ = 0.017, p = 0.899; interaction effect: *F*_(1,26)_ = 3.979, p = 0.057). (E) The procedure of virus injection and the CFC test (n = 6–9/group). (F) The freezing time of mice during contextual fear conditioning (repeated two-way ANOVA, main effect of intervals: *F*_(3,72)_ = 162.1, p < 0.0001; main effect of vehicle vs. JZL195: *F*_(3,24)_ = 0.095, p = 0.962; interaction effect: *F*_(9,72)_ = 0.701, p = 0.706). (G) The freezing time of mice during Test 1 (two-way ANOVA, main effect of gene type: *F*_(1,24)_ = 0.239, p = 0.630; main effect of vehicle vs. JZL195: *F*_(1,24)_ = 2.702, p = 0.113; interaction effect: *F*_(1,24)_ = 2.337, p = 0.139). (H) The freezing time of mice during Test 2 (two-way ANOVA, main effect of gene type: *F*_(1,24)_ = 0.009, p = 0.926; main effect of vehicle vs. JZL195: *F*_(1,24)_ = 18.360, p = 0.0003; interaction effect: *F*_(1,24)_ = 1.743, p = 0.199; Sidak’s post hoc test of WT-Vehicle vs. WT-JZL195: p = 0.040, CB1R^H−KO^-Vehicle vs. CB1R^H−KO^-JZL195: p = 0.002).

As shown in Figure 6, the CB1R protein could not be detected in the hippocampus only, while CB1R expression in the other brain areas was not affected. The timeline of this experiment is shown in Figure 7A. As shown in Figure 7, wild-type mice spent more time in the open arms of the EZM 24 h after the drug injection test (Figure 7B), while CB1R^H−KO^ mice spent even less time in the open arms. We did not observe any difference in the distance traveled or the frequency of entering the open arms between these two mouse lines (Figures 7A and 7C). CB1R^H−KO^ mice showed freezing time comparable to that of wild-type mice during contextual fear conditioning (Figure 7F) and the short-term memory test (Test 1, Figure 7G). On the other hand, both wild-type mice and CB1R^H−KO^ mice had longer freezing times in the recent memory test (Test 2, Figure 7H). These results suggested that JZL195 may contribute to memory consolidation and recall in a noncannabinoid receptor-dependent manner, but hippocampal CB1R may mediate the anxiolytic effect induced by JZL195.

## Discussion

Endocannabinoids have been well documented to play crucial roles in learning and memory.^14^^,^^25^ In the literature, controversial results regarding the intervention effect of endocannabinoid-degrading enzymes have been collected thus far. Knockout of MAGL, an enzyme that predominantly degrades 2-AG, was reported to promote spatial learning and memory in the water maze task and the long-term plasticity of CA3-CA1 synaptic transmission.^15^ However, in another report, both JZL195 and JZL184 (a selective inhibitor of MAGL) were observed to impair the performance of mice in the water maze.^16^

The influences of MAGL and FAAH deficits on fear conditioning also seem inconsistent across studies. Direct injection of AEA into the nucleus accumbens (NAc) core slightly reduced the freezing time during the contextual fear memory retrieval task,^26^ and systemic administration of URB597, a selective inhibitor of FAAH, impaired fear memory recall.^27^ However, when the time point of drug injection was moved backward to the start of the learning phase, the systemic administration of URB597 enhanced contextual fear memory acquisition, and this effect can be inhibited by JZL185 delivered into the prefrontal cortex or the ventral hippocampus.^28^ If the clue was set as a tone, the systemic administration of JZL184 did not affect fear conditioning, but the fear memory was too strong to be extinguished.^24^ In addition, the enhancement of tone-cued fear memory recall induced by JZL184 but not URB597 was also reported.^29^ Despite these different reports, all the aforementioned positive outcomes achieved from drug administration or genetic manipulation are CB1R dependent. Few studies have been designed to probe the potential role of CB2R, although it was still reported as an important player in contextual fear memory^8^ and a key mediator of JZL184’s effects on fear memory.^29^ In the present study, we found that JZL195, a dual inhibitor of MAGL and FAAH, enhanced the performance of mice in the contextual memory retrieval task. This effect disappeared 30 days later when the mice were returned to Context A as a remote memory test. We also found that the dual inhibition of MAGL and FAAH did not affect the consolidation and recall of tone-cued fear conditioning, as mice did not show much more freezing time in Context B in the presence of training tone. This result suggested that JZL195 may take effect in a context-related brain area such as the hippocampus, which has been identified as a crucial brain area taking part in the regulation of contextual memory and fear memory, as evidenced by significantly increased c-Fos-expressing neurons after contextual fear memory recall. In addition, our data also reveal the change in hippocampal LFPs, which, especially theta (4–7 Hz) oscillation, have been demonstrated, by ample evidence, to be the key neuronal mechanism of learning and memory.^30^^,^^31^^,^^32^^,^^33^ We found here that the theta oscillation power was higher than the basal level 2 h after JZL195 injection. Since the critical period of memory consolidation after acquisition ranges from 2 to 6 h,^34^ the increase in theta oscillation in our study may suggest that JZL195 has the potential to promote memory consolidation.

In this study, a decreased frequency of mEPSCs was observed, which indicated a presynaptic change triggered by JZL195. Conversely, the increased amplitude of mEPSCs is often regarded as a postsynaptic change resulting from upregulated glutamate receptor subunits, mainly α-amino-3-hydroxy-5-methyl-4-isoxazole-propionicacid receptor (AMPAR), and enhanced density of dendritic spines. All these alterations may contribute to high levels of neuronal activity and potential long-term potential (LTP) that underlie efficient memory retrieval. The effect of endocannabinoid (eCB) related drugs is often considered to work through CB1R or CB2R and therefore the following intracellular pathways. However, in the present study, we did not succeed in illustrating the necessity of either CB1R or CB2R for the effect of JZL195 because both the antagonists and knockout mice failed to inhibit the increased freezing. This result suggests that the other newly found receptors (e.g., TRPV1 and GPR55) may take the place of CB1R and CB2R in the contextual fear memory task.

In addition to contextual fear memory, we also found that the injection of JZL195 at a dose of 20 mg/kg produced an anxiolytic effect in the EZM test, which was mediated by CB1R, and this effect was abolished by genetic hippocampus-specific deletion of CB1R. The potential therapeutic effect of eCB has been discussed for decades; however, the dose and the injection time point do matter when applying eCB-related reagents such as JZL184, URB597, PF-3845, and JZL195. For JZL195 specifically, 3 mg/kg administration produced an antidepressant effect in an major drepressive disorder model 24 h after the last injection^35^ but was reported as pro-depression when directly introducing mice into the force-swimming test 3 h after injection,^36^ as was 20 mg/kg in the same report. In an anxiety-like behavioral test, when JZL195 was administered at 10 mg/kg and 2 h before the test, mice showed no such alteration in anxiety state either following acute stress or not.^37^ JZL184 exhibited anxiolytic effects at various doses 3 h after injection.^38^ Consistent with one of the aforementioned reports,^35^ we found in this study that JZL195 produced an anxiolytic effect at a dose of 20 mg/kg when applied 24 h before the EZM test. This result also contradicts some other reports that showed the complex effect and underlying mechanism of JZL195. When CB1^H−KO^ mice were involved, JZL195 even had a pro-anxiety effect. A possible mechanism is that the anxiolytic effect was mediated by CB1Rs expressed on glutamatergic axon terminals,^39^ as we revealed a decrease in the frequency of mEPSCs. Once CB1R is removed, accumulated eCB, particularly AEA, activates TRPV1 receptors, which then increase anxiety-like behavior.^40^

In summary, we established that JZL195 significantly promotes memory consolidation and retrieval in a foot shock-based fear memory task. However, due to the discrepancies among behavioral test paradigms or other unknown reasons, including pharmacological and multi-brain area interaction factors, further investigations are probably needed before the precise and detailed role that the ECS plays in different phases of memory formation can be elucidated. Moreover, we also reported a hippocampal CB1R-dependent anxiolytic effect of JZL195. Nonetheless, our study suggests the feasibility of targeting endocannabinoid-degrading enzymes for the clinical treatment of mental disorders.

### Limitations of the study

In this study, we found that the dual inhibitor of FAAH and MAGL could enhance the performance of mice in the contextual fear memory task and the activity of the hippocampus. This is the first time that JZL195 was found to benefit a typical type of memory at a high dose. Although this study suggests the possibility of JZL195 contributing to the treatment of mental disorders, more specific experiments involving disease models should be conducted to identify the potential of JZL195. The subsequent mechanism underlying the anxiolytic effect of JZL195 should be investigated in detail.

## STAR★Methods

### Key resources table

**Table:** 

REAGENT or RESOURCE	SOURCE	IDENTIFIER
**Antibodies**		

Rabbit monoclonal anti-c-Fos	Cell Signaling Technology	Cat#2250; RRID: AB_2247211
Rabbit polyclonal anti-CB1R	Frontier Institute	Cat#CB1-Rb-Af380; RRID: AB_2571591
Donkey anti-rabbit, AlexaFluor 647 conjugated	JacksonImmunoResearch	Cat#711-605-152; RRID: AB_2492288

**Bacterial and virus strains**		

AAV2/9-CMV-Cre-EGFP	Taitool	Cat#S0231-9

**Chemicals, peptides, and recombinant proteins**		

JZL195	MedChemExpress	Cat#HY-15250
AM281	Sigma-Aldrich	Cat#A0980
NESS0327	Cayman Chemical	Cat#10004184
SR144528	MedChemExpress	Cat#HY-13439

**Experimental models: Organisms/strains**		

Mouse: C57BL/6J	Jax	Cat#000664
Mouse: *Cnr1*^*flox/flox*^	This paper	N/A

**Software and algorithms**		

EthovisionXT	Noldus	https://www.noldus.com.cn/ethovision-xt/
VideoFreeze	Med-associates	https://med-associates.com/product/videofreeze-video-fear-conditioning-software/
pClamp 10.5	Molecular Devices	https://www.moleculardevices.com/products/axon-patch-clamp-system/acquisition-and-analysis-software/pclamp-software-suite
OpenEphys GUI	OpenEphys	https://open-ephys.org/gui
Spyking-circus	Yger et al.^41^	https://github.com/spyking-circus

### Resource availability

#### Lead contact

Further information and requests for resources and reagents should be directed to and will be fulfilled by the lead contact, Jing Han (jhan2012@snnu.edu.cn).

#### Materials availability

This study did not generate any new materials.

#### Data and code availability

• Data reported in this paper will be shared by the lead contact upon request.
• This paper does not report original code.
• Any additional information required to reanalyze the data reported in this paper is available from the lead contact upon request.

### Experimental model and study participant details

#### Wild-type and transgenic animals

Male C57BL/6J mice aged 10-16 weeks were used in this study. The number of animals used in each experiment is stated in the figure legends or corresponding *results* sections. We do not reuse mice among tests unless otherwise stated.

*Cnr1*^*flox/flox*^ mice were constructed by Biocytogen (Beijing, China) as previously described^42^. Two loxP sites were inserted at each end of the first exon of the CB1R gene (encoded by the Cnr1 gene). We performed genotyping using PCR with the following primers. Forward: ACTGGACAGCTCATCCCTTG and reverse: AAGTCAATGGTCTTGCATGGATCT (301 bp *Cnr1*^*flox/flox*^, 244 bp wild type).

#### Hippocampus-specific deletion of CB1R

To confirm whether hippocampal CB1R mediates the effect of JZL195, we introduced a genetically modified mouse line. We constructed a mouse line with a hippocampus-specific deletion of CB1R. We injected Cre-carried AAV into the hippocampal area. Therefore, the Cre/loxP system will work specifically in the hippocampus and result in the deletion of Cnr1, the encoding gene of CB1R (the *Cnr1*^*flox/flox*^*::CMV*^*Cre*^ mouse line will be described as CB1R^H-KO^ mouse in the manuscript, in which “H-KO” represents hippocampus-specific knockout).

##### Virus injection

*Cnr1*^*flox/flox*^ mice were anesthetized with 1.5∼2% isoflurane and then fixed stereotaxically. AAV2/9-CMV-Cre-EGFP (volume: 300 nl/side, titer: 2.0E+12 vg/ml) was injected into the hippocampus (AP: -1.9, ML: ±1.2, DV: -1.6 mm) at a velocity of 50 nl/min. The syringe was held there for 5 minutes and then dropped off slowly. As the experimental control, wild-type mice were injected with Cre-carried virus as the same as the *Cnr1*^*flox/flox*^ mice (so called). To obtain efficient deletion of CB1R, mice were moved to the behavioral procedure 1 month after virus injection.

#### Ethical statement

Mice were acquired from the laboratory animal center of the Key Laboratory of Modern Teaching Technology, Ministry of Education, Shaanxi Normal University. Mice were reared in a constant temperature and humidity environment (22±1°C, 30-40% RH) on a scale of 4-5 per cage, and the indoor day and night cycle was controlled by a fixed intensity light source (Turn on time 8:00-20:00). Each mouse was acclimated for 1-2 min, and this procedure lasted for three days before the behavioral experiments. Mice were fed *ad libitum* and euthanized using CO_2_ after finishing all tests. The experimental protocols described here were approved by the Animal Ethics Committee of Shaanxi Normal University.

### Method details

#### Drug preparation

JZL195 (20 mg/kg) was dissolved in DMSO and diluted in sunflower oil. AM281 (3 mg/kg), NESS0327 (0.3 mg/kg), and SR144528 (3 mg/kg) were dissolved in DMSO and diluted in a solution containing 10% Tween-80 and 80% saline. All drugs were delivered through intraperitoneal injection (i.p.). The final concentration of DMSO in the working solution was kept below 10% to avoid potential biotoxicity.

#### Behavioral tests

##### Contextual fear conditioning (CFC)

Contextual fear memory was examined using CFC as previously described.^43^ Mice were introduced into the fear conditioning box (MED-VFC-USB-M, Med associates, USA) and allowed to explore the box freely for 4 minutes (cumulative freezing time during this phase was set as the baseline). Then, three foot shocks (0.75 mA, 2 s) were delivered at 4, 6.25, and 8.5 minutes after the beginning of the experiment. Ninety seconds after the last foot shock, the mice were transferred back to the home cage.

###### Short-term memory test of contextual fear memory

Mice were returned to the conditioned box (Context A) 2 hours after conditioning and were monitored for 5 minutes.

###### Long-term memory test (also defined as the Recent test in the manuscript)

Mice were returned to the conditioned box (Context A) 24 hours after conditioning and were monitored for 5 minutes.

###### Remote memory test (also defined as Remote test in the manuscript)

Mice were returned to the conditioned box (Context A) 30 days after conditioning and were monitored for 5 minutes.

##### Auditory fear conditioning (AFC)

To examine cue-related fear memory, another important type of fear memory that is often thought to be expressed and regulated by the cortex and amygdala, an AFC protocol was performed. Mice were introduced into the fear conditioning box and received foot shocks as described in the Contextual fear conditioning (CFC) section. To build a connection between a specific cue and the foot shock, a 60 s-long tone was presented just before each foot shock. Cued fear memory was tested 24 hours after conditioning. Mice were introduced into a box (Context B) that had a completely different context from the conditioning box (Context A) and were monitored for 5 minutes.

Video was collected and analyzed using VideoFreeze, a commercial software provided by Med-associates.

##### Elevated zero maze (EZM)

EZM was used to test whether mice were anxious. The EZM used in this study was made of organic glass (at a height of 60 cm), with an inner diameter of 51.8 cm and an outer diameter of 65 cm. The closed arms of the EZM are separated by two 15 cm-high organic glasses, the outer one of which is opaque. After a 15-minute habituation, mice were put into the EZM and allowed to freely move for 10 minutes. The video was collected and analyzed using Ethovision XT. The distance that mice traveled, the time, and the frequency that mice entered into the open arms were compared between groups to evaluate anxiety-like behavior.

#### Immunofluorescence of c-Fos and CB1R

Immunofluorescence experiments were performed to evaluate the c-Fos expression induced by the contextual memory test and to verify the deletion of CB1R in the hippocampus. Mice were sacrificed and perfused with saline. The brain was taken and immediately immersed in precooled 4% PFA and kept at 4°C overnight. The PFA was then replaced with 30% sucrose solution in 1x PBS and kept at 4°C until the brain sunk into the bottom of the vial. Forty-micron coronal sections were collected using a cryostat microtome. After washing with PBS, the slices were blocked with blocking buffer containing 10% normal donkey serum, 0.3% Triton-X 100, and 89.7% PBS for 2 hours at room temperature. Then, we incubated slices with primary antibodies and secondary antibodies in a 24-well plate. After the slices were pasted, slides were mounted with antifade reagents. Hippocampal images were collected using a Zeiss M2 microscope. c-Fos-positive cells were automatically counted in ImageJ software.

#### Electrophysiology

##### *In vitro* experiment - Slice preparation

Mice were anesthetized with urethane and then decapitated. The brain was quickly removed from the skull and immersed in precooled sucrose-based cutting solution (in mM, 225 sucrose, 2.5 KCl, 1.25 NaH_2_PO_4_, 26 NaHCO_3_, 11 D-Glucose, 5 L-Ascorbic Acid, 3 Sodium Pyruvate, 7 MgSO_4_·7H_2_O, 0.5 CaCl_2_). After being fixed on a metal pallet, the brain was cut into 300-μm slices. Slices were then collected and incubated in artificial cerebrospinal fluid (ACSF) containing (in mM): 122 NaCl, 2.5 KCl, 1.25 NaH_2_PO_4_, 26 NaHCO_3_, 11 D-Glucose, 2 MgSO_4_·7H_2_O, 2 CaCl_2_ equilibrated with 95% O_2_-5% CO_2_ at 28°C for at least 1 hour before recording.

##### Whole-cell recording of miniature excitatory postsynaptic current (mEPSC)

mEPSCs were recorded in CA1 pyramidal neurons to measure excitatory transmission from CA3 to CA1. When performing patch-clamp, ACSF contained 100 μM PTX, and 1 μM TTX was perfused constantly to keep cells healthy. The k-based intracellular solution contained 120 K gluconate, 20 KCl, 10 HEPES, 10 phosphocreatine Na salt, 2 ATP Na salt, 0.4 GTP Na salt and 2 MgCl_2_, pH 7.35, were used to make a whole-cell recording. Pyramidal neurons in dorsal CA1 were visually identified and clamped. After a stable whole-cell recording was made, the membrane potential was clamped at -70 mV. We then acquired mEPSCs in gap-free mode. Five-minute-long continuous data were saved and analyzed using pClamp.

##### *In vivo* experiment - Electrode implantation

Mice were anesthetized with 2% isoflurane and held on a stereotype (RWD, China). Sixteen-channel microwire array electrodes (KD-MWA, KedouBC, China), a 4x4 array consisting of 25 μm NiTi wire spaced 200 μm, were slowly inserted into the mouse brain. Four small nails were first inserted into the skull, with a ground wire presoldered onto one of them. The electrode was left at the dorsal hippocampus (AP: -1.9, ML: -1.2, DV: -1.5 mm), and then dental cement was used to fix it onto the skull.

##### Data acquisition

Mice were introduced to the recording arena at least one week after surgery. During the day, the electrode on the mouse skull was connected to the OpenEphys acquisition board through an Intel head stage. OpenEphys GUI was used to visualize and save electrical signals. Mice were allowed to freely move inside a home cage-like arena for at least 20 mins. Only data acquired during the last 10 min were saved and then analyzed by Python-based software and a customized MATLAB script (described below).

##### Single unit isolation

Spikes were detected and divided into single units using the Spyking-circus.^41^ Continuous binary raw data (sampling rate: 30 kHz) were imported and filtered using a bandpass butter filter at a cutoff value of 300 Hz. Movement artifacts were removed by subtracting medians over all channels. The spike detection threshold was set at a 6-fold median absolute deviation (MAD), while spikes beyond 10-fold MAD were abandoned as artifacts. Templates used to detect spikes were automatically generated, and the width of the templates was manually designated as 2 ms. Clustering was also automatically processed using a density-based cluster method, and clusters with a Bhatta distance less than 0.5 were merged into a single cluster. Neurons meeting the following criteria were excluded from the following analysis: (1) spikes with refracting period violations smaller than 1 ms, accounting for more than 1.5% of total spikes, and (2) total frequency lower than 0.2 Hz (probably noise) or larger than 20 Hz (probably fast-spiking interneurons).

##### Local field potential (LFP) power spectrum

Raw data were loaded into MATLAB and bandpass filtered at 0.5-150 Hz. The power spectrum of LFP was analyzed using the MATLAB function “Signal Analyzer”. The relative power of the specific θ oscillation (4-12 Hz) was calculated per channel.

### Quantification and statistical analysis

Data are presented as the mean±SEM in all figures included in this manuscript. To compare mean values between the two groups, in terms of the normally distributed data and equal standard deviation, Student’s t test was performed in GraphPad software. Otherwise, the Mann‒Whitney test was performed instead. In terms of the normally distributed data and equal standard deviation, one-way or two-way ANOVA was performed in GraphPad software followed by Sidak’s or Tukey’s post hoc test to compare mean values among three or more than three groups. Otherwise, the Kruskal‒Wallis test followed by Dunn’s post hoc test was performed instead.

## Supplemental information

Document S1. Figures S1‒S3
