# Supplementary material for: Inhibition of cannabinoid degradation enhances hippocampal contextual fear memory and exhibits anxiolytic effects
Source: iScience. 2024 Jan 15;27(2):108919. doi: 10.1016/j.isci.2024.108919 (PMC10839683; doi:10.1016/j.isci.2024.108919)
Supplement: Document S1. Figures S1‒S3 [file mmc1.pdf]

iScience, Volume *27*

Supplemental information

Inhibition of cannabinoid degradation enhances hippocampal contextual fear memory and exhibits anxiolytic effects

Jinming Zhang, Junmin Zhang, Ruiqi Yuan, Wenxin Han, Yuan Chang, Lingyang Kong, Chunling Wei, Qiaohua Zheng, Xingchao Zhu, Zhiqiang Liu, Wei Ren, and Jing Han

Supplemental information

Figure S1-S3.

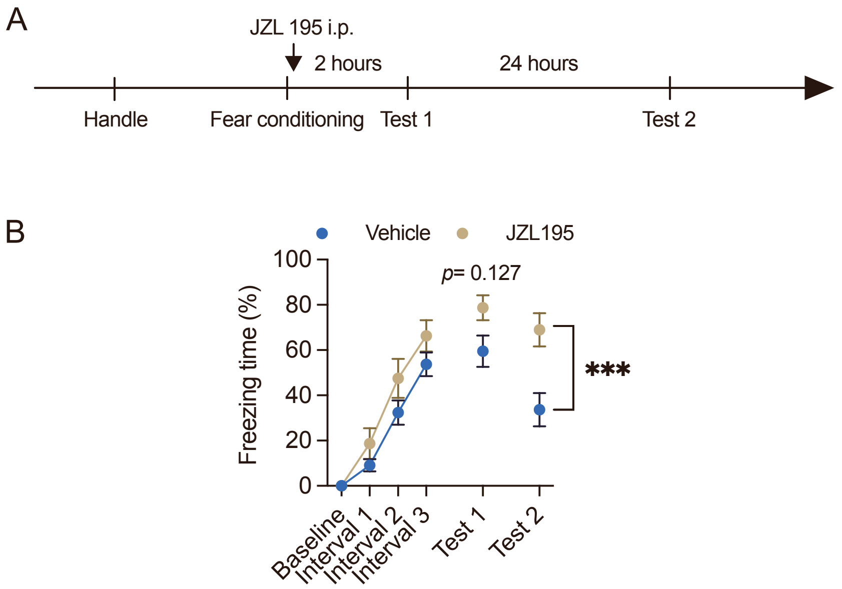

Supplemental Figure S1, related to Figure 1 - JZL195 treatment has no influence on short term fear memory

(A) The procedure of short term fear memory test ($n_{Vehicle}$=12, n_{JZL195}=10).

(B) The freezing time of mice during contextual fear conditioning, Test 1 and Test 2 (Two-way ANOVA, the main effect of time: $F_{(5,95)}$=76.280, p<0.0001; the main effect of Vehicle vs. JZL195: $F_{(1,19)}$=6.379, p=0.021; the interaction effect: $F_{(5,95)}$=3.597, p=0.005; Sidak's posthoc test of Vehicle vs. JZL195 at Test 1: p=0.127, at Test 2: p=0.0003).

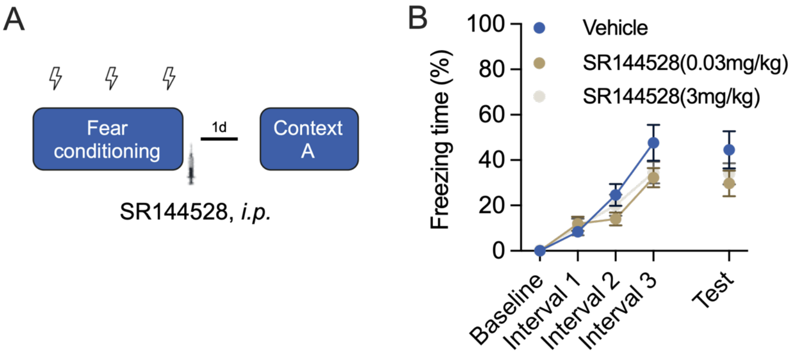

Supplemental Figure S2, related to Figure 7 - The effect of SR144528 on contextual fear memory consolidation.

(A) The procedure of contextual fear conditioning (n=10/group).

(B) Different doses of SR144528 show no effect on the freezing time of mice (Two-way ANOVA, the main effect of intervals: $F_{(4,108)}$=60.380, p<0.001; the main effect of doses: $F_{(2,27)}$=1.489, p=0.244; the interaction effect: $F_{(8,108)}$=1.622, p=0.127; Sidak's posthoc test of Vehicle vs. SR144528 (0.03mg/kg) at Test: p=0.055, Vehicle vs. SR144528 (3mg/kg) at Test: p=0.226, SR144528 (0.03mg/kg) vs. SR144528 (3mg/kg) at Test: p=0.781).

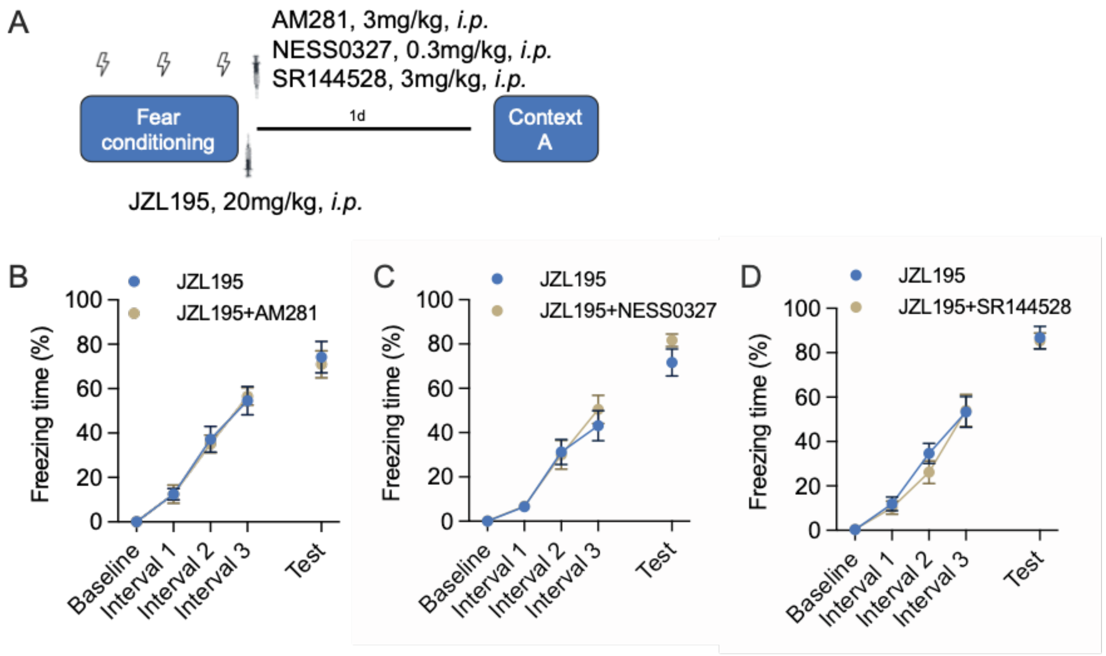

Supplemental Figure S3, related to Figure 7 - Neither CB1R nor CB2R suppressed the promoted contextual fear memory induced by JZL195.

(A) The procedure of behavioral test.

(B) The freezing time of mice during fear conditioning and contextual test (Two-way ANOVA, the main effect of intervals: $F_{(4,68)}$=119.00, p<0.0001; the main effect of JZL195 (n=10) vs. JZL195+AM281 (n=9): $F_{(1,17)}$=0.022, p=0.885; the interaction effect: $F_{(4,68)}$=0.136, p=0.969; Sidak's posthoc test of JZL195 vs. JZL195+AM281 at Test: p=0.993).

(C) Freezing time of mice during fear conditioning and contextual test (Two-way ANOVA, the main effect of intervals: $F_{(4,68)}$=123.900, p<0.0001; the main effect of JZL195 (n=10) vs. JZL195+NESS (n=9): $F_{(1,17)}$=0.585, p=0.455; the interaction effect: $F_{(4,68)}$=0.818, p=0.518; Sidak's posthoc test of JZL195 vs. JZL195+NESS0327 at Test: p=0.512).

(D) Freezing time of mice during fear conditioning and contextual test (Two-way ANOVA, the main effect of intervals: $F_{(4,72)}$=180.900, p<0.0001; the main effect of JZL195 (n=10) vs. JZL195+SR (n=10): $F_{(1,18)}$=0.229, p=0.634; the interaction effect: $F_{(4,72)}$=0.515, p=0.725; Sidak's posthoc test of JZL195 vs. JZL195+SR144528 at Test: p=0.999).
